# A Public Health Analysis of the Relationship Between Loneliness, Isolation, and Dementia

**DOI:** 10.1093/geroni/igab046.918

**Published:** 2021-12-17

**Authors:** Christina Victor

**Affiliations:** Brunel University London, Uxbridge, England, United Kingdom

## Abstract

Loneliness and isolation are now characterised as major public health problems largely because of reported associations with negative health outcomes including dementia. We adopt a public health perspective and review the relationship between loneliness/isolation and dementia focussing on how these concepts are defined, measured, and reported. We identified community based longitudinal studies which measured loneliness/isolation at baseline and dementia at follow up (minimum 12 months) published up to February 2021. We identified 12 papers for loneliness and 15 for isolation which demonstrated substantial heterogeneity in how exposure (loneliness/ isolation) and outcome (dementia) were measured and reported. For example, dementia was measured in 5 different ways: death, hospitalisation, clinical diagnosis, dementia screening tools or cognitive function. Evidence to support a relationship between loneliness/isolation and dementia is inconclusive largely because of this methodological heterogeneity. Using consistent exposure and outcome measures is a prerequisite for determining the health consequences of loneliness and isolation.

